# Examining Socioeconomic and Computational Aspects of Vaccine Pharmacovigilance

**DOI:** 10.1155/2019/6576483

**Published:** 2019-02-19

**Authors:** Vasiliki Soldatou, Anastasios Soldatos, Theodoros Soldatos

**Affiliations:** ^1^Department of Computer Science and Biomedical Informatics, University of Thessaly, Greece; ^2^Department of Business Administration, School of Business, Athens University of Economics and Business, Greece; ^3^Independent Researcher, Heidelberg, Germany

## Abstract

**Background:**

Vaccine pharmacovigilance relates to the detection of adverse events, their assessment, understanding, and prevention, and communication of their risk to the public. These activities can be tedious and long lasting for regulatory authority scientists and may be affected by community practices and public health policies. To better understand underlying challenges, we examined vaccine adverse event reports, assessed whether data-driven techniques can provide additional insight in safety characterization, and wondered on the impact of socioeconomic parameters.

**Methods:**

First, we integrated VAERS content with additional sources of drug and molecular data and examined reaction and outcome occurrence by using disproportionality metrics and enrichment analysis. Second, we reviewed social and behavioral determinants that may affect vaccine pharmacovigilance aspects.

**Results:**

We describe our experience in processing more than 607000 vaccine adverse event reports and report on the challenges to integrate more than 95500 VAERS medication narratives with structured information about drugs and other therapeutics or supplements. We found that only 12.6% of events were serious, while 8.97% referred to polypharmacy cases. Exacerbation of serious clinical patient outcomes was observed in 8.88% VAERS cases in which drugs may interact with vaccinations or with each other, regardless of vaccine activity interference. Furthermore, we characterized the symptoms reported in those cases and summarized reaction occurrence among vaccine-types. Last, we examine socioeconomic parameters and cost-management features, explore adverse event reporting trends, and highlight perspectives relating to the use and development of digital services, especially in the context of personalized and collaborative health-care.

**Conclusions:**

This work provides an informative review of VAERS, identifies challenges and limitations in the processing of vaccine adverse event data, and calls for the better understanding of the socioeconomic landscape pertaining vaccine safety concerns. We expect that adoption of computational techniques for integrated safety assessment and interpretation is key not only to pharmacovigilance practice but also to stakeholders from the entire healthcare system.

## 1. Introduction

The goal of pharmacovigilance is early detection of adverse events (AEs) and appropriate and timely response in order to minimize negative effects to the health of individuals. There is a number of national and international postmarketing surveillance systems, including the VigiBase [1] by the World Health Organization (WHO) International Drug Monitoring Programme, the US FDA Adverse Event Reporting System (FAERS; formerly AERS) [2, 3], and the European Eudravigilance [4] network.

The Vaccine Adverse Event Reporting System (VAERS) [5] is an early warning system specific to US-licensed vaccine safety. Different sites allow browsing VAERS data (e.g., [6] or [7]) or track changes in reports over time (e.g., [8]). Vaccine adverse events (VAEs) can be electronically submitted to VAERS (e.g., [9, 10]) that accepts all reports, including both cases of vaccination errors and cases for which it is unclear whether a vaccine caused the VAE. Therefore, VAE assessment and risk management are continuously integral to the vaccine pharmacovigilance process and computational methods help validate possible false signals that may be attributed to confounding factors or to reporting bias.

These complex activities can be tedious and long lasting for regulatory authority scientists, primarily due to the fact that large parts of the data come in free-text. This kind of format makes the act of performing efficient systematic analysis difficult. To alleviate the situation in VAE safety detection and prediction, advanced text-mining, and other techniques are employed for feature extraction, semantics, and rule deduction (see [11, 12]), while disproportionality metrics are utilized as the main signal detection standard [13]. The application of such data mining methods requires however a decision regarding the sufficient definition of a threshold of identified signal strength above which a potential relationship should be considered interesting for further investigation [14]. Another consideration is country-specific names and availability of vaccines around the world [15], as well as the normalization of VAE symptoms. Different controlled terminologies have been developed for the classification of AE reactions such as MedDRA [16], or CTCAE [17], and the vaccine-specific OVAE [18]. Such formally structured ontology annotations are created and maintained by experts but may also come with drawbacks, such as the lack of semantic or textual definitions [19].

While extensive work exists that copes with similar challenges regarding the general AE study and characterization, data-driven approaches have been shown to be powerful in AE detection and prediction [20]. Such approaches include the blending of information from omics data, social media, and electronic registries and employ a variety of statistical methods, machine learning and data mining techniques [20]. These developments are expected to affect also vaccine discovery and development, as well as vaccination campaign and vaccine safety monitoring [21].

In this regard, rational mechanism based assessment of pharmacovigilance statistics plays important role for vaccine safety scientists [22]. While several systems-biology efforts to combine molecular information with phenotypes exist (e.g., [23–27]), they rely primarily on side-effect information coming from labels, on omics and literature data. Systematic use of complementary information for safety detection enhancement (e.g., [28, 29]) from other big data repositories, such as electronic registries available throughout the healthcare system [20–22], remains somewhat underutilized. VAERS is one such augmented source, providing electronic VAE data that capture real-world scenarios regarding vaccine uses, combinations, phenotypes, and conditions not studied in clinical-trials, and also includes information for many more patients.

Additionally, despite the fact that vaccine immunization mechanisms are at large different than the main therapeutic-intervention biology in the presence of a disease, a number of cases have indicated the possibility of vaccine-drug interactions [30, 31]. In some cases, this may be attributed to drug metabolism changes following immunization, which may occur, for example, when vaccines are administered in patients, likely treated with multiple drugs. Previous work has shown that incorporation of chemical and biological reference data can help assess the biological plausibility of such drug related AE signals [22, 28, 32].

In this work, we analyze VAERS to provide examples of such computational challenges and highlight the importance of structuring VAE data (Table 1). We emphasize that annotation of VAE content (e.g., of the therapeutic agents reported) should be done in a way that allows easier, augmented, or novel exploration of relationships, such as of patient indications and preconditions, of polypharmacy (drug and drug-classes) and of molecular (targets, metabolizing-enzymes) interactions. To evaluate the extent that such strategies are feasible, we initially report on our experience of augmenting VAEs with drug and molecular data and then elaborate on the content of VAERS. Specifically, we present results regarding different drug interference scenarios that could be identified in VAEs using this approach and explore the prevalence of reactions occurring in those cases. Next, we assess benefits and limitations of automatically summarizing vaccine safety profiles from these data, a central challenge in the process of vaccine safety detection. Finally, to better comprehend the landscape pertaining to vaccine safety, we also examine behavioral determinants and socioeconomic parameters that may affect vaccine pharmacovigilance (Table 2).

## 2. Materials and Methods

To understand intricacies underlying VAE data, we reviewed VAERS content. First we annotate drug mentioning in VAE cases and, then, follow a dual analysis approach:

- We explore the extent of drug interference in VAEs and also assess the prevalence of reactions in those cases
- We evaluate whether it is possible to automatically generate comprehensive vaccine safety profiles from these data

**Figure 1:**
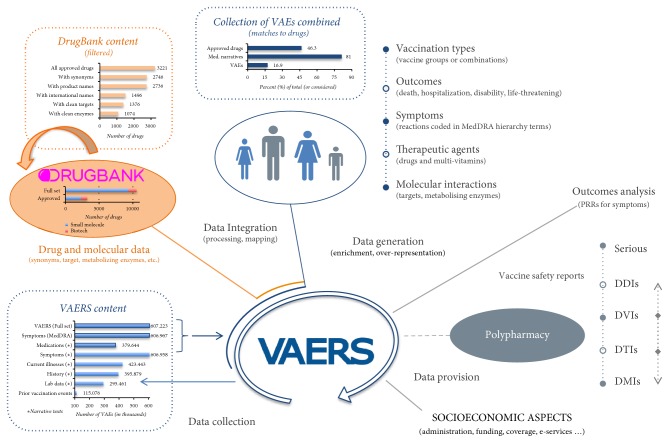
*VAERS review approach*: we process public VAE reports from VAERS to identify serious events, annotate polypharmacy cases, and highlight those in which drugs may interact with each other regardless of vaccine activity interference. Among other content, VAERS contains data regarding adverse vaccine incidences, respective outcomes, reported symptoms, information about vaccine administration, diagnostic laboratory data, preexisting and current conditions, and narratives about (prescription, or not) medications that the vaccine-recipient was taking at the time of vaccination. Next to disproportionality measures, we utilize enrichment-analytics to compare reaction incidence between cases with and without drug interference. We also summarize VAE reports to provide vaccine safety profiles.

To perform both of these tasks we first had to expand VAE content with drug and/or molecular information—Figure 1 summarizes our analysis approach.

### 2.1. Data Integration

We extracted VAE data from VAERS and drug and molecular information from DrugBank [33–35]. VAERS was used to extract symptoms coded in MedDRA terms and patient outcomes (see Supplementary Methods). We defined as serious VAEs for which “death,” “hospitalization,” “life threatening,” or “disability” events were reported. From DrugBank we extracted known drug-drug interactions, drugs known to affect the therapeutic efficacy, or the VAE risk or severity of vaccinations, as well as drug targets and metabolizing enzymes (see Supplementary Methods). Using these data, we identified polypharmacy events (namely, VAE-cases to which more than one drug mapped) and defined four levels of possible drug interference:

- Cases with drugs known to interact with each other (DDIs)
- Cases with drugs known to affect the therapeutic efficacy, or the VAE risk, or severity of vaccinations (DVIs)
- Cases with potential interactions between drugs due to perturbation of the same targets (DTIs)
- Cases with drugs sharing the same metabolizing enzymes (DMIs)

To identify drugs mentioned in VAERS, we followed a previously employed approach [22] and matched the nonstructured medication narratives that are reported in VAERS in free-text format, by using a drug dictionary compiled from DrugBank (see Supplementary Methods).

### 2.2. Statistical Characterization

To explore the relative association of symptoms and outcomes to different VAE-sets we employed two main computational techniques.

- * Disproportionality Metrics*. First, we used the proportional reporting ratio (PRR), an established measure of disproportionality in pharmacovigilance. PRR gives an indication for the relative congruence of pairwise entity relations as based on their cooccurrence in (sub-)sets of VAE data and was calculated using the approach described by van Puijenbroek et al. [36]. For a VAE set (S) and an event (E) the PRR metric as shown in Table 3.
- * Enrichment Analysis*. To examine the overrepresentation of an event E (symptom or outcome) within the set of VAEs (S) with drug interference, we followed an approach similar to [19, 37] where the PRR metric was used as enhancement factor (i.e., ratio describing the relative representation of E in S).
- * Vaccine Safety Profiling*. Similarly, the PPR score was used to quantify the extent of each symptom-vaccine association with respect to the occurrence of each of its parts in VAERS. In specific, in this case an event (E) represented occurrence of a symptom, tested against the VAE-set of each vaccine type (S).

In all cases, Fisher's exact test (two tailed) was used to determine the statistical significance of each observation. Last, we defined minimum occurrence in at least ten VAEs as reasonable threshold to consider a relationship meaningful.

### 2.3. Software

This work utilized PostgreSQL 9.6 for storage, Python for calculations, and Java for additional programming tasks.

## 3. Results

We processed 607223 VAE reports from VAERS that contained 218 vaccine-names and 10169 symptoms (Additional File 1). The dataset was integrated together with drug and molecular information from DrugBank by processing 95397 vaccine medication narratives.

### 3.1. The Combined Collection

DrugBank contained mainly small molecule therapeutics (i.e., low molecular weight drugs produced by chemical synthesis), but also agents manufactured in or extracted or semisynthesized from biological sources. These included medical agents (e.g.,* Cetuximab* or* Lepirudin*), nutritionals, and other supplements (from, for example, fruits and foods like* avocado*,* banana*,* grapefruit*,* garlic*, and* watermelon* to* tobacco leaf* or* fish oil*), as well as vaccine records. We therefore did not identify DVI medications ourselves (e.g., by considering immunomodulating agents, or other drug classes) and extracted interactions with vaccines directly from DrugBank's own list. We also extracted synonyms for 3221 approved drugs and compiled a dictionary, which consisted of 61516 nonredundant names for 3218 of those drugs.

Using this dictionary, we matched drug names to VAERS medication narratives. We noticed that some were quite noisy (e.g., containing abbreviations, information about manufacturer, dosage, medication schedule, patient history, dramatic complaints, etc.), while others mentioned cosmetic or nutrition agents and other supplements. Characteristically we found potentially 13732 such VAEs referring to (multi-) vitamin use, just by annotating the mentioning of “VIT” in a narrative. Overall, by matching 77314 medication narratives (81%), we successfully annotated 102487 (16.9%) VAEs. Of those, 98963 (96.6%) linked to 1491 (46.3%) approved drugs.

### 3.2. Processing VAERS Narratives

**Figure 2:**
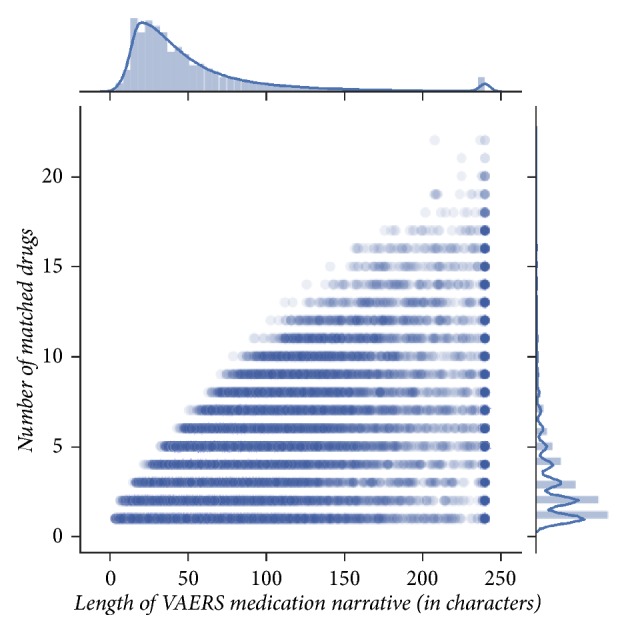
*Drug name mapping performance*: we contrast the number of drugs that were matched in a narrative against its length (sample Pearson correlation coefficient* r *= 0.69 reflects the effect of noise introduced by longer “dirty” narratives that did not only contain medication names). We notice that the larger the narrative's length was, the more drugs matched—most matches happened between narratives that were smaller in size.

A large proportion of VAEs (37.5%) had no medication narratives, while some were not informative. For example, the top five most frequently occurring phrases included “NONE” (103490 VAEs), “NO OTHER MEDICATIONS” (72735 VAEs), “UNKNOWN” (30951), “UNK” (23455 VAEs), and “CONCOMITANT DRUG(S) NOT REPORTED” (4428 VAEs). In our mapping approach we did not account for typographical and spelling errors that appeared in some narratives (e.g., “%DEXAMETHAZON%,” “AVENDOL, TREZEDONE AND DESIPREANINE,” or “LEXAPOR TRAZADONE”). We also did not consider advanced regular expressions, types of drug classes, or semantics that would help in few cases to avoid both false negatives and false positives. For example, the phrase “LATANOPROST0.005% EYE DROPS” was falsely not mapped, while* Lipitor* was falsely mapped to “LIPITOR - NOT PRESENTLY TAKING. ZEDIA - REPLACED LIPITOR. MYCARDIS. ASPRIN.” Last, one systematic cause for false negatives in our approach was the decision to not include in the dictionary names of mixtures (i.e., with >1 ingredients). However, although the mentioning of such medications (like* Exforge*,* Augmentin*,* Atripla*, or* Adderall*) in narratives would not be captured, our choice for strict name definitions favored subsequent unambiguous DTI and DMI identification. Despite these shortcomings, such a simplistic mapping approach performed well (Figure 2). This can be attributed to the smaller size of most narratives, as well as their little redundancy.

However, VAERS does not contain only medication narratives. Because mining unstructured free text can be challenging, especially regarding biomedical nomenclature [19], VAERS provides a large list of explanations for commonly used abbreviations in the context of its VAES which can be used to accommodate more advanced techniques. Overall, VAERS narratives contain potentially much more information-rich content regarding indications/conditions, laboratory results, or even family history and allergies that await mining.

### 3.3. VAE Symptoms and Severity

**Figure 3:**
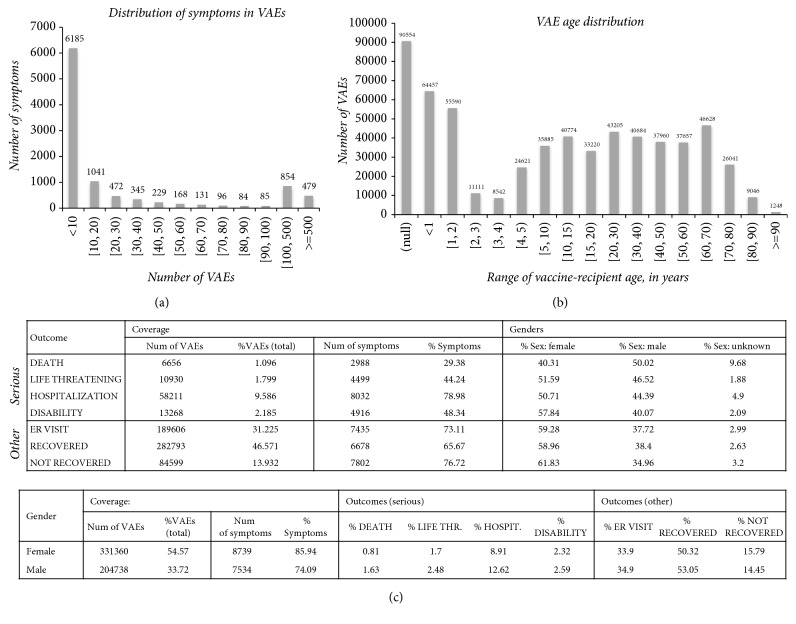
*Vaccine content demographics*: (a) distribution of symptoms among VAEs—most symptoms appeared in few cases; more than one symptom might be linked to a single VAE; (b) VAE age distribution—VAERS contains events that refer to all ages; 15% of VAEs did not have available age information; we assumed that VAE incidence among smaller ages is more likely to reflect regular/routine vaccination events, contrary to older age VAEs that might refer to patients suffering from (sometimes, severe) medical conditions; (c) outcome-gender reporting in VAERS—46.6% of cases reported in VAERS recovered, while 12.5% of VAEs were serious; differences in gender distribution invite for more thorough examination of underlying circumstances or of possible differences in the function of male/female metabolism effects.

We then focused on VAE symptoms—VAERS contained 10169 symptoms coded in terms coming from 23 different MedDRA versions. Most symptoms (60.8%) appeared in <10 VAEs (Figure 3(a)). Similarly, most VAEs (96%) also linked to <10 symptoms, while 256 had none. Surprisingly, the third most frequent symptom was the term “NO ADVERSE EVENT” appearing in 49830 VAEs (8.2%). In addition, the whole VAERS dataset held only 76234 serious VAEs (12.6%), which indicates that, in spite of its size, the largest part of VAERS refers to minor events.

Furthermore, a large amount of VAEs refer to elderly (Figure 3(b)), probably medically more vulnerable and prone to suffer from severe health problems and receive multiple medications. Also, many other symptoms, coreported with the “NO ADVERSE EVENT” term, referred to drug-related events (e.g., “MEDICATION ERROR,” “DRUG TOXICITY,” or “DRUG ADMINISTRATION ERROR”). We wondered, then, to what degree might drugs be accountable for VAEs or influence their severity?

### 3.4. Polypharmacy and Drug Interference in VAEs

Our vaccine content indicated that, of the 98963 VAEs that had been mapped to approved drugs, more than half (55%) linked to multiple drugs (>1; vitamins not considered). Of these 54454 polypharmacy VAEs (8.97% of all VAEs), 6172 (11.3%) were serious and accounted for 8.1% of the total serious VAEs. Overall, 8620 reactions (84.8%) were reported with 76122 serious VAEs (Figure 3(c)), an observation that highlights how these events are of great concern.

We therefore investigated polypharmacy cases further and assessed the distribution of symptoms and of serious outcomes among VAEs with higher likelihood of drug interference. We defined those drug interference VAEs as polypharmacy cases that contained known DDIs, or potential DTI- or DMI-inferred interactions. We also included cases for which drug interference might not be attributed to polypharmacy alone and looked for VAEs mentioning at least one DVI drug.

**Figure 4:**
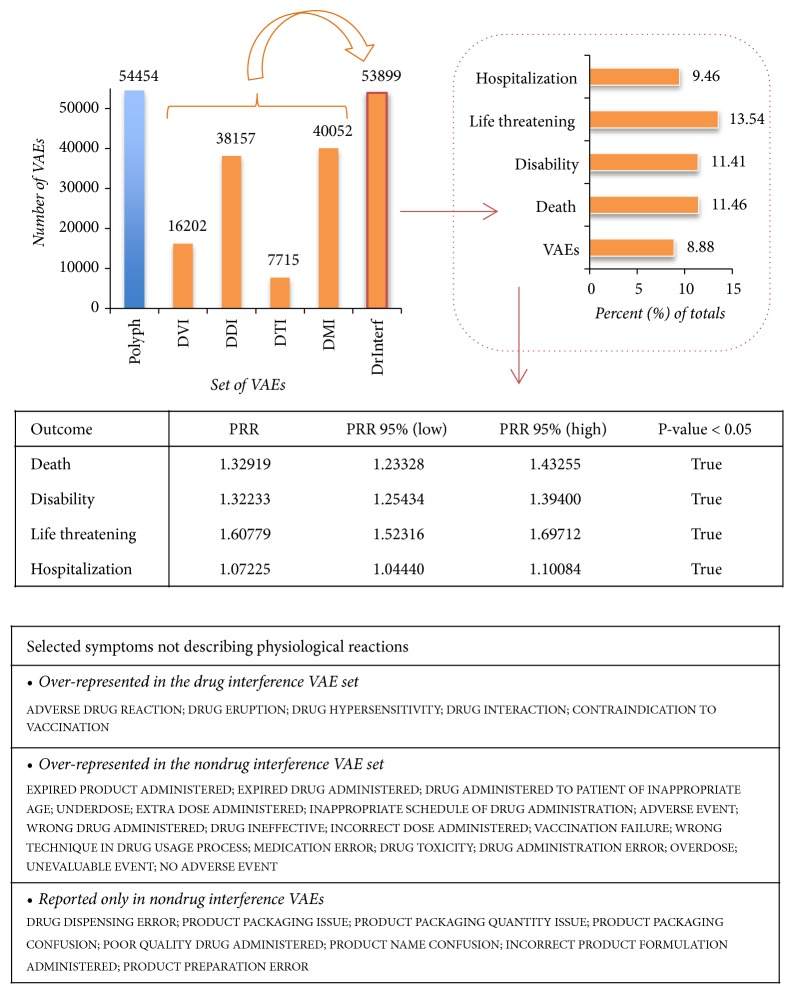
*Analysis of VAEs with drug interference*: we identified drug interference VAEs (DrInterf) as polypharmacy cases (Polyph) that included known DDIs, or potential DTI- or DMI-inferred interactions. Drug interference VAEs included also cases that mentioned at least one DVI agent. Serious outcome occurrence was increased in this set of VAEs, when compared with the rest of VAERS (middle table: PRR highlights overrepresentation of death, disability, and life threatening events). To analyze drug-related symptoms in this set we focused on reaction terms that did not describe phenotypes (lower table). Such symptoms (overrepresented in this set of VAEs) were terms describing events attributed to drug interference. Occurrence of drug-related reactions in the “opposite” non-DrInterf set (e.g., “DRUG TOXICITY”) can be explained from the fact that it also contains VAES characterized by drug occurrence or polypharmacy. This denotes that drug-induced risks are not limited to the DrInterf set, despite the observed exacerbation among them. The analysis also revealed prevalence of events reported in VAERS that did not refer to VAEs (see “NO ADVERSE EVENT” symptom) in cases with decreased drug interference risk—in those VAEs, remaining terms indicate that many cases could be attributed to incorrect product use and preparation, or administration errors.

In total, we identified 53899 such possible drug interference VAEs (8.88% of all VAERS): interestingly, this set contained 16202 VAEs with DVIs alone, already a significant proportion of VAERS (2.7%). The set contained also 38157 VAEs with DDIs and 7715 and 40052 VAEs with DTIs and DMIs, respectively. Notably, manifestation of serious outcomes is exacerbated among those VAEs (Figure 4). Importantly, these findings suggest that many serious cases reported in VAERS may be falsely attributed to vaccines.

### 3.5. Drug-Induced and Other Errors in VAEs

Moreover, we analyzed the 5460 symptoms mentioned in drug interference VAEs (Additional File 2). Of those, 406 appeared only in those VAEs but with too few mentioning (<3 VAEs). From the rest, 1533 were found to be statistically significant: among those, 433 symptoms were overrepresented (PPR>=2 and >10 VAEs) and 53 were underrepresented (PRR<=0.5 and >10 VAEs) in the set. From the remaining 4709 symptoms that were not mentioned in drug interference VAEs, only 351 had >10 VAEs. We manually examined each set of symptoms and found several terms that did not describe phenotypes or reactions (Figure 4).

These results verified higher occurrence of drug-induced events in drug interference VAEs, but they also revealed a range of errors for the remaining set of VAEs that could be attributed to vaccine administration or to medical and therapeutic procedures. We believe that, irrespective of whether it was iatrogenic or patient factors underlying those cases, their occurrence calls for improved immunization practices and raises the issue of education to highlight awareness for both medical personnel and patients.

### 3.6. Automated Vaccine Safety Profiling Intricacies

Next, we sought to characterize the relationship between vaccinations and symptoms, as reported in VAEs. The dataset held 218 vaccination names for ninety vaccine types (Additional File 3). Vaccine names referred to brand names (e.g., “*DTP (TRI-IMMUNOL)*”), while vaccine types referred to groups of similar vaccinations (e.g., “*DT*” for “*diphtheria and tetanus toxoids, pediatric*” or “*DTP*” for “*diphtheria and tetanus toxoids and pertussis vaccine*”). In some VAEs multiple vaccine names for the same type were mentioned (e.g., due to VAERS historical changes or case data updates), as well as vaccines of more than one type. In this work we did not examine covaccination occurrence as many times different vaccines may be administered simultaneously depending on the immunization program. We also did not examine vaccination dose, route, or site information due to many missing values. We processed symptom occurrence at the level of vaccine types, as organised in VAERS. This also helped to decrease the dataset of candidate associations to 132093 symptom-to-vaccine-type combinations (Additional File 4).

By filtering out nonsignificant associations, our analysis narrowed down the set by 91.5% and 79% with respect to the total candidate relationships and symptoms, correspondingly. Characteristically, ten vaccine types were mentioned in too few VAEs and had no significant associations. Our threshold of choice was maybe too strict, favoring thus confidence in cooccurrences with larger numbers of VAEs (Table 4).

We chose to validate our results by looking at the safety profile produced for BCG (Table 5), a vaccine used for protection against tuberculosis, which in turn is the most vaccine-preventable cause of death worldwide (Supplementary Figure 1). The profile included reactions related to fever and vomiting, irritations at the injection site, tuberculosis, and infections, as well as lymphadenopathies and breathing difficulties, all consistent with multiple resources [38–41]. While evaluating the higher occurrence of pneumonia, death, and urinary issues, BCG use in cancer immunotherapy came to our attention. Intravesical BCG is, for example, an effective treatment of superficial bladder cancer [42, 43]. We found three vaccination names grouped under the* BCG* category in VAERS, namely, “BCG (MYCOBAX),” “BCG (NO BRAND NAME),” and “BCG (TICE).” However, without further examination of additional VAE information (e.g., current or preexisting conditions) it could not be explicitly determined whether BCG was involved as a tuberculosis preventive vaccination or as a bladder cancer therapeutic. Indeed, in a recent study BCG VAEs had to be manually checked to identify their safety profiles [44]. This also emphasizes the importance of structuring and annotating VAE data, as an approach to enable quicker and more efficient mining.

### 3.7. Socioeconomic Perspectives

Our results indicate that vaccines are overall safe—indeed, immunization is one of the most cost-effective public health interventions to date, saving millions of lives [45]. Yet, according to UNICEF, one in seven children worldwide did not receive the required third dose of DTP in 2016 [46]. DTP's child vaccination rates for some OECD countries were not much better [47], when DTP is one of the vaccines with the largest world coverage according to the WHO (Supplementary Figure 1). This also emphasizes that resources should be dedicated more efficiently to benefit world population and help avoid vaccine-preventable deaths.

Such health effects can translate also into positive economic results, as vaccination can provide significant savings by avoiding direct and indirect costs associated with the treating of diseases and possible long-term disabilities [48]. One US study estimated that every dollar spent on childhood vaccination could save 3$ from a payer perspective and 10$ from a societal perspective [49]. In Europe, the recent financial crisis has put tremendous economic pressures leading to arbitrary cuts in healthcare budgets—an average of 9% of gross domestic product was allocated to national healthcare, while only 3% of this was dedicated to prevention [50].

### 3.8. Research and Development

**Figure 5:**
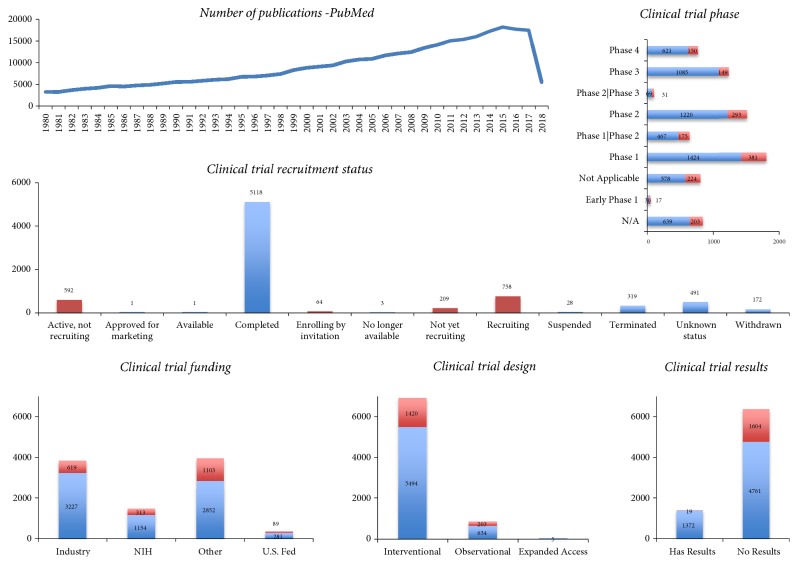
*Immunization is an active field of study*: a number of publications in PubMed (upper, left) and of clinical trial features from clinicaltrials.gov (remaining plots) mentioned “vaccine” or “vaccination” or “immunization.” On a historical note, another 67932 publications have been published prior to 1980 with the oldest one dating back up to 1819. Red color refers to 1623 vaccine trials that are “running” defined as those that have recruitment status “Active, not recruiting,” “Enrolling by invitation,” “Not yet recruiting,” or “Recruiting” (middle). Of those, 527 (32%) refer to conditions mentioning the terms neoplasm, cancer, tumor, melanoma, glioma, leukemia, or neuroblastoma. Upper, right: many current trials study the effectiveness of vaccines. Notably, there are as many Phase III trials (last premarketing stage) as in Phase IV (post-FDA approval stage). Lower, left: 49.6% of vaccine related trials involve industry funds. Category “other” denotes academic and research organizations, hospitals, military/defense centers, as well as non-US institutions and authorities, governments, universities, and international and nonprofit organizations. In comparison, VAERS mentions vaccines from 39 manufacturers. Lower, middle: most vaccine trials tend to be interventional (participants are assigned to groups) rather than observational (often retrospective). Lower, right: unfortunately, many trial results are not made available, hindering thus transparency and reproducibility.

While these developments represent potentially important consequences for healthcare systems and the health of citizens, they also encourage investing in research and development (Figure 5). Different* in silico* tools exist, to aid and assist researchers in this complex vaccine discovery and design process [21]. Characteristically, out of 7756 vaccine-related clinical trials listed in clinicaltrials.gov [51], we found that 21% are currently running (or about to start). Next to “traditional” context (allergies, pregnancy and newborn safety, tuberculosis, zika, malaria, anthrax, measles, meningitis, polio, influenza, rabies, etc.), current vaccine trials include conditions ranging from HIV- and HPV-infections to diabetic or metabolism (renal/liver) related complications.

Indeed, it is expected that major role in the future of vaccine pharmaceutics will play revenue potential from vaccination of adolescents and adults, as opposed to sales from the vaccination of children that drove this market in the past. This is somewhat reflected by VAERS age demographics (Figure 3(b)) and is also in accord with vaccine clinical trial activity. In specific, we found that one third of the currently running vaccine trials study vaccines in the context of cancer therapeutics.

Expecting the returns of a long, risky, and expensive discovery process, industry drives big part of clinical trial development, while a variety of other stakeholders participate with the incentive to develop new, cheaper, and safer vaccines. Vaccinomics play a special role in this process, enabled by the widespread diffusion of high-throughput omics disciplines, technologies, and approaches in the field of vaccinology [21]. Part of the challenge is also economic, as governments and insurers would like to reduce unnecessary or avoidable postmarketing costs. For example, at least half of the cases reported in VAERS include some form of public, military, or private spending (Supplementary Figure 2).

### 3.9. Public Trends and Collaborative Health Strategies

Another aspect influenced by management and administration policies is public opinion. One such example is the public concern caused by the 2009 swine flu vaccine shortage and its direct impact on vaccine safety perception. This is elegantly demonstrated by the peak in Google searches for “Vaccine safety” in October 2009 [52] and its direct correlation with “swine flu shot” (Pearson correlation* r* > 0.98 by Google Correlate [53]). The observed increase in VAERS reports during this vaccine safety discussion (Supplementary Figure 2) suggests that news and other media may affect the rate of AE reporting, too. In comparison, FAERS contains only 24042 AEs related to immunization procedures and vaccines, and its overall vaccine content growth seems to not have been affected by this event alone.

On the positive side, (pre-)school vaccine administrations are more due to government mandates and support, rather than result of economic or public opinion incentive. There are, however, considerable political, organizational, and logistical challenges to the delivery of such large scale programs. Challenges include funding, vaccine supply and distribution, staff capacity and workload, anxiety and distress to students, and consent and reach of parents [54]. We find that informing parents and children about the feasibility and results of early vaccination can help engage the public in health studies worldwide. Such collaborative health strategies would enable not only the recording of more detailed vaccination result statistics but also the efficient addressing of observed complications.

### 3.10. Digital Services and Personalized Mobile Apps

Production and consumption of personalized health apps may be one way to enable such new collaborative models. Several studies in mobile use have demonstrated that active patient participation can benefit vaccination programs [55], facilitate VAE reporting [56], and provide access to trustworthy vaccine information [57]. We anticipate that use and development of digital services can promote coordination and collaboration between multiple stakeholders in health including individuals, schools, pharmacies, medical personnel, hospitals, states, authorities, and postmarketing surveillance programs.

However, we find that this market has not yet reached its potential. Studies show that vaccination coverage in mobile apps follows neither the growth of media use nor the related advancement of technological features [58]. Recent work studying the benefits from vaccine-related mobile apps reported availability of less than 250 such services [59]; some of them are government endorsed [60]. We examined ourselves a few such applications to find a large diversity in functionalities, target-users, and providers—typically, we found that services provided by authorities primarily aim to reach health professionals and have more downloads. Overall, main functionalities include (but are not limited to) information about vaccines, handling and storage of personal or family health records, immunization schedules, and reminders. However, we pinpointed that provision of geographic based information (such as variation in local vaccination plans or outbreak news), country-specific download availability, and language representations are aspects that may significantly limit reachable audiences and long term uptake. These observations highlight also challenges in coordinating international regulatory efforts, as well as difficulties in collecting and harmonizing vaccine information universally and for any location.

### 3.11. Game Theory and Education Strategies

Education also plays important role—while digital technologies may serve well as a mechanism to empower users and increase participation in the immunization process, they have also revolutionized our ability to educate ourselves. Reasonably, key part of vaccine information relates to safety and precaution issues regarding contraindications and allergies. However, it becomes increasingly necessary to communicate the need to make vaccinations as planned, to all members involved in each society.

This is because several reasons exist that may have undermined vaccine importance. First, disease eradication occurring in some places may mask the cost-benefit relationship for an individual, family, or community. Then, vaccine credibility may have been weakened by the familiarization of the public with circumstantial profit-driven industry practices. Furthermore, this does not help adequately limit a dilemma that some doctors perhaps may often face: to take the responsibility that a vaccine will have no side effects, and this, regardless of the fact that it is not absolutely certain it will provide the desired immunity.

The answer is not univocal. Certainly safety concerns should be communicated, but not at the expense of how general immunization is perceived. Game theory models show that it is “herd immunity” rather than self-interest that can help outweigh the risk of infection through vaccination [61, 62]. Voluntary vaccination policies should therefore communicate risks but also emphasize the overall group benefit. This does not contrast modern personalized health interests, but it rather highlights the need to provide objective communications and results derived from data examined on the basis of thorough evidence-based criteria.

## 4. Discussion

Vaccines have historically improved quality of life. Optimizing earlier capture of safety and error risks can help leverage vaccine value and provide higher levels of health quality. However, to accelerate modern pharmacovigilance insight requires strategies that are able to provide more mechanistic (causative) explanations of observed safety concerns [22]. Structuring of real-world AE data and integration with additional sources of information helps towards this direction by allowing broader and more specific analytics [20–22]. Importantly, it provides regulatory, pharmaceutical, and pharmacovigilance scientists with the critical ability to not only systematically perform retrospective epidemiological studies, but also transparently assess any potentially involved biomolecular rationale that may underlie emerging observations [22].

VAERS is one such source of VAE observations, but its content must be dealt with caution when interpreted, as these data alone cannot be used to determine a cause-effect relationship between a vaccination and an AE [13]. One such example is the false association of autism with vaccination [63], a signal captured also by our automated vaccine safety profiling. Also, VAE narratives can be dirty and need to be mined carefully. Furthermore, VAERS data may contain biases and may be influenced by public response to media attention. Last, VAERS contains only VAEs and symptom incidence is not normalized with respect to overall population vaccine consumption data. Statistical signals and derived incidence rates should therefore be subjected to further analysis and be confirmed in controlled studies [64].

In the context of this work, VAERS was used for hypothesis generation—we assessed the extent of polypharmacy-induced risks and found that prevalence of serious outcomes is higher in VAEs with more definitive risk of drug interference. This also suggests that many serious VAEs may be falsely attributed to vaccines.

Facilitating such data-driven techniques for broader analytics is one factor for determining strategies to improve safety [20–22]. We also reviewed features related to socioeconomic parameters. We examined aspects related to cost-management (vaccine administration facility and fund source) and vaccine development (research and clinical trials), explored AE reporting trends (including demographics and public opinion effects), and assessed perspectives relating to the use and development of digital services to help raise awareness and empower patient and physician engagement in immunization practices. Indeed, enormous databases, such as immunization registries and surveillance systems, can be mined to capture data concerning vaccination effectiveness, coverage rate, and its determinants [21].

As mobile technology continues to rapidly evolve, we expect that mobile apps offer the potential to improve the quality of information residing in immunization evaluation programs, facilitate harmonization between individuals, health care providers and public health systems, and may help reduce vaccine hesitancy—a hesitancy that may perhaps be attributed to several factors. Some of those include the fact that reduced disease infection rates have contributed to increased perception of vaccine-induced risks, the easy spread of news through modern media, and the lack of education about immunization, what vaccines are or how they work. In some ways, fear of disease became fear for the vaccine—some might say that vaccines have been the victims of their effectiveness.

Game theory models explain that this is an understandable behavior, reasonably driven by individual self-interest. They do, however, also provide “selfish” arguments towards performing the “altruistic” act of vaccination that governments should harvest. Voluntary vaccination programs should incentivize and promote community protection and highlight the expectation to save millions of lives. The economic cost estimate to this synergistic individual-population benefit plays also an important role to make the right decisions on vaccination policy.

Our work also calls for the development of more refined algorithms that will allow for novel data streams to be combined and mined. Big data play key role in this perspective, which have contributed and are expected to continue contributing toward facilitating the discovery, development, production, and delivery of more rationally designed vaccines and immunization practices [21]. Moving toward more tailored and personalized vaccine design and administration, big data solutions can help effectively integrate and harmonize together many precious resources and databanks that are highly heterogeneous [20, 21]. What is also required is a set of reliable benchmarks tailored specifically for safety detection and prediction approaches by enabling comparisons and data exchange to be based on fair and equivalent reference [19, 22, 37]. Further prospects inspired by potential big data driven applications include also better determination and communication of vaccine efficacy, safety and side effects, vaccination policy effectiveness, and addressing vaccine literacy and hesitancy issues [21]. We find that, in the context of personalized health, such developments will help nourish key aspects to capitalize upon for more collaborative health-care strategies, such as shared decision-making opportunities and better-informed self-management.

In the future we plan to advance and automate our approach for reviewing VAERS and to systematically provide services for researchers and the public. We expect to benefit from updated drug, molecular, and VAERS content, as well as considering also information about foods, fruits, and nutritionals or supplements. To address data extremities we want to enhance our analysis with extended synonym dictionaries and ontologies and hierarchies for reaction categories and drug classes. Last, we also plan to expand our approach by testing against known vaccine and drug side effects, examine indications and subpopulation susceptibility, and investigate the influence of combinatorial drug and vaccine occurrences in the incidence of specific symptoms.

## 5. Conclusions

We envisage that our work will provide a broad understanding of the socioeconomic and computational challenges underlying vaccine pharmacovigilance, as well as an attractive framework for improving the performance of safety signal detection algorithms. We demonstrated that structuring AE data and integration of molecular information can potentially provide additional insight into existing approaches, but also an easy way to quickly and systematically produce safety hypotheses. Importantly, it enables a standardized approach to the development of more objective analytics and promotes public domain transparency. We find that key to any healthcare system stakeholder is the adoption of integrated safety assessment and interpretation strategies, not only to avoid adverse incidence and preventable costs, but importantly to accommodate opportunities for advancing community health, personal awareness, and quality of life.

## Figures and Tables

**Table 1:** *Computational challenges in analyzing VAE data*: vaccine pharmacovigilance activities can be tedious and long lasting for regulatory authority scientists. Integration with additional resources (e.g., molecular data) may provide new possibilities to augment VAE analytics. Using this approach, we reviewed the extent of polypharmacy and drug interference cases in VAERS.

Computational challenges	Description	Type
Reported VAE content	VAE reporting systems may also contain cases for which it is unclear whether a vaccine caused the VAE. Also follow-up is not always possible. VAE data alone cannot be used to determine a cause-effect relationship between a vaccination and an AE.	Qualitative
Large parts of the data come in free text	Examples include narratives regarding patient medications, laboratory results, or disease history. Advanced text mining or other techniques can be employed for feature extraction, semantics, and rule deduction.	

Mining unstructured content	One way to structure VAE data is by mapping content to organized dictionaries and/or hierarchies of therapeutic agents (e.g., vaccines and drugs) or phenotypic manifestations (e.g., diseases, medical conditions, symptoms, side-effects, and reactions). These tasks can be complicated, affected by several factors such as the nonstandard nature of the used nomenclature (e.g., country specific names), nonrelevant content, quality of the entity recognition method used, completeness of the underlying dictionary/hierarchy, annotation coverage, and appropriate representation/detection of relationships.	Quantitative
Automated signal detection	While disproportionality metrics are utilized as the main signal detection standard, there is no sufficient (or universal) definition of a threshold for identified signal strength above which a potential relationship should be considered interesting for further investigation. Also, detected signals may sometimes refer to false positive associations.	

**Table 2:** *Socioeconomic parameters affecting vaccine pharmacovigilance*: we examined aspects related to vaccine pharmacovigilance activities (detection, assessment and understanding, prevention, and communication) from social, behavioral, and financial perspectives.

Socioeconomic challenges	Description	Pharmacovigilance aspect			
		Detection	Assessment / understanding	Prevention	Communication
VAE reporting	VAERS contains only VAEs and symptom incidence is not normalized with respect to overall population vaccine consumption data.	X	X		X

Vaccine development	Cancer vaccine therapeutics and vaccination of adolescents and adults is an important part of current research focus and clinical trial activities.		X		

Cost management	While it is beneficial for the healthcare systems to prevent unnecessary or avoidable costs, political, organizational and logistical challenges may significantly hinder the delivery of large-scale vaccine administration programs.			X	X

Digital services	While use and development of digital services can promote the coordination of healthcare stakeholders, systemize real world data collection, help raise awareness, and empower both patient and physician engagement in immunization practices, relevant mobile phone services that are provided currently are largely maintained by authorities, primarily aiming to reach mainly health professionals.	X		X	X

Collaborative health policies	Shared and better-informed decision-making is key for improving international efforts in harmonizing worldwide vaccine management and information.	X		X	X

Public opinion	VAE data may contain biases and may be influenced by public response to news and media attention.		X		X

Vaccine hesitancy	While key part of vaccine information relates to safety and precaution issues, the easy spread of news, lack in education, and reduced disease infection rates have contributed to increased perception of vaccine-induced risks. It becomes increasingly necessary for voluntary vaccination programs not only to communicate these risks but also to emphasize the benefits of vaccination for the population in order to incentivize and promote community protection.			X	X

**Table 3:** Proportional reporting ratio (PRR): the PRR metric is defined as the value of *a(c+d)/c(a+b)*, based on the following contingency matrix.

VAE cases	*Event * **(E)**	*Not * **E**	Totals
*Set * **(S)**	*a*	*b*	*a + b*
*Not * **S**	*c*	*d*	*c + d*
Totals	*a + c*	*b + d*	*N = a + b + c + d*

**Table 4:** *Summary of vaccine safety profiles*: our analysis reduced large part of the original candidate set of associations. Our threshold criteria required statistical significance, PRR values to be larger than one, and the signals to be observed in more than ten VAEs. This is reflected by the increased average values observed for relationships included in the summarized profiles. These contain some extreme values that, for example, may occur when almost all incidences of a symptom appear in VAEs of one vaccine type.

Totals	Unprocessed set	Profile summary

Symptom-to-vaccine combos	132093	11287
Vaccine types	90	80
Symptoms	10169	2133

Averages	Unprocessed set	Profile summary

VAEs per vaccine type	10614.2	11938.25
Symptoms per vaccine type	1467.7	141.09
PRR score (symptom-vaccine type)	-	11.37
% symptom occurrence per vaccine type	0.3	1.34

**Table 5:** *BCG reaction profile*: our approach allows producing easily and systematically comprehensive vaccine safety profiles. BCG findings were all verified in labels and other educational material. The profile included also non-reaction terms like ‘POLYMERASE CHAIN REACTION', a laboratory procedure used for rapid diagnosis of tuberculosis. BCG was reported in 421 VAEs only, indicating reduced VAE risk—also, percentage representation of symptoms' occurrence with BCG should be interpreted with respect to the context of the vaccine's overall use and not only as reported in VAERS.

Reaction	Num of VAEs (total)	Num of VAEs (BCG)	PRR	%BCG's VAEs
BOVINE TUBERCULOSIS	15	14	20178.7	3.32542
TUBERCULOSIS	32	12	864.8	2.85036
DYSURIA	500	20	60.1	4.7
LYMPHADENITIS	355	13	54.8	3.1
POLLAKIURIA	416	14	50.2	3.3
RESPIRATORY RATE INCREASED	566	17	44.6	4
INJECTION SITE ABSCESS	1032	12	16.9	2.8
POLYMERASE CHAIN REACTION	1344	13	14.1	3.1
HAEMOGLOBIN NORMAL	1258	11	12.7	2.6
DEATH	2766	20	10.5	4.8
HAEMATOCHEZIA	2058	11	7.7	2.6
LYMPHADENOPATHY	7759	33	6.2	7.8
PNEUMONIA	3310	13	5.7	3.1
COUGH	13116	36	3.9	8.6
LABORATORY TEST ABNORMAL	6327	15	3.4	3.6
IRRITABILITY	7757	12	2.2	2.9
INFECTION	13014	19	2.1	4.5
CHILLS	19150	24	1.8	5.7
DIARRHOEA	16105	20	1.8	4.8
PYREXIA	100453	107	1.5	25.4
VOMITING	28847	30	1.5	7.1

## Data Availability

The data used to support the findings of this study are included within the supplementary information files.

## Abbreviations

AE: Adverse event
BCG: Bacillus Calmette-Guerin
CTCAE: Common Terminology Criteria for Adverse Events
DDI: Drug-drug interaction
DMI: Drug-metabolizing enzyme interaction
DT: Diphtheria and tetanus toxoids, pediatric
DTI: Drug-target interaction
DTP: Diphtheria and tetanus toxoids and pertussis vaccine
DVI: Drug-vaccine interaction
HIV: Human immunodeficiency virus
HPV: Human papilloma virus
FDA: Food and Drug Administration
FAERS: FDA Adverse Event Reporting System
MedDRA: Medical Dictionary for Regulatory Activities
OVAE: Ontology of Vaccine Adverse Events
PRR: Proportional reporting ratio
VAE: Vaccine adverse event
VAERS: Vaccine Adverse Event Reporting System
WHO: World Health Organization.

## Supplementary Materials

Supplementary 1 File name and format: Additional_File_1.txt. Title: Occurrence of reactions in VAERS. Description: Tab separated file containing the number of VAEs in which each reaction was reported.

Supplementary 2 File name and format: Additional_File_2.txt. Title: Symptom over-representation in drug interference VAEs. Description: Tab separated file containing the name of each reaction (event_symptom_reaction), the total number of VAE cases the reaction was reported in (event_count), the total number of cases that the reaction was reported in drug interference VAEs (event_count_in_drug_interference_vaes), respective contingency matrix 1 values (a, b, c, d), the respective PRR score and 95% confidence interval (prr, prr_95ci_min, prr_95ci_max), and the respective Fisher's two-tailed test p-value and if it is <0.05 (pvalue_fisher_exact_test_two_tailed, significant_by_fisher_exact_test).

Supplementary 3 File name and format: Additional_File_3.txt. Title: Vaccine occurrence in VAEs. Description: Tab separated file listing the vaccination type code (vax_code), the number of VAE cases reporting this vaccine type (num_vaes_type), vaccination names reported in VAERS for each vaccine type (vax_name), and the number of VAEs that the respective vaccine name was reported in (num_vaes_name).

Supplementary 4 File name and format: Additional_File_4.txt. Title: Vaccine reaction profiles. Description: Tab separated file containing vaccine types (vax_type), number of VAEs that the vaccine type was mentioned in (num_vaes_vax_type), reaction names co-reported with the respective vaccine type (symptom_reaction_event), total number of cases that the reaction was mentioned in (num_vaes_symptom), number of VAE cases that the respective vaccine type and reaction were co-reported (num_vaes_vax_n_event), contingency matrix 1 values (a, b, c, d), respective PRR score and 95% confidence interval (prr, prr_95ci_min, prr_95ci_max), the respective Fisher's two-tailed test p-value and if it is <0.05 (pvalue_fisher_exact_test_two_tailed, significant_by_fisher_exact_test), and percent (%) of the vaccine type's VAEs that the reaction was co-reported (percent_event_in_vax_vaes).

Supplementary 5 File name and format: Additional_File_5.pdf. Title: Supplementary methods and figures. Description: Supplementary method details and Figures 1 and 2.
