# Supplementary material for: Examining Socioeconomic and Computational Aspects of Vaccine Pharmacovigilance
Source: Biomed Res Int. 2019 Feb 19;2019:6576483. doi: 10.1155/2019/6576483 (PMC6399563; doi:10.1155/2019/6576483)
Supplement: Supplementary 5 — File name and format: Additional_File_5.pdf. Title: Supplementary methods and figures. Description: Supplementary method details and Figures 1 and 2. [file 6576483.f5.zip › Additional_File_5.pdf]

Supplementary material

Methods: To analyze VAERS we expanded VAE content with drug and/or molecular information:

- **Structuring medication narratives**: To identify drugs in VAEs we relied on the medication narratives accompanying each case. These come in non-structured or free text format, as recorded in the VAERS form, and report information about any prescription or non-prescription drugs that the vaccine-recipient was taking at the time of vaccination. We therefore created a drug dictionary, the names of which were then matched against these narratives. The dictionary was compiled from DrugBank [1],[2],[3] records - to avoid semantic ambiguity, the names 'baby', 'patch' and 'same' were not used and synonyms were required each to describe only one drug-entry and be more than three characters long. Similarly, we also did not process narratives with less than four characters (like 'FLU', 'O2', 'WET' 'MOM', 'PUB', 'PPD', 'YES', 'UNK' etc.) and excluded noisy narratives that appeared in many cases (e.g., 'NO OTHER MEDICATIONS' or 'CONCOMITANT DRUGS NOT REPORTED').
- **Polypharmacy and molecular integration**: Having structured medication narratives, we could enrich VAE data with information about drugs and the molecular perturbation of their targets and metabolizing enzymes. First, we identified polypharmacy events, namely VAE-cases to which more than one drugs mapped. Then, we defined four levels of possible drug interference: (a) cases with drugs known to interact with each other (DDIs), (b) cases mentioning drugs known to affect the therapeutic efficacy, or the VAE risk or severity of vaccinations (DVIs), (c) cases with potential interactions between drugs due to perturbation of the same targets (DTIs), and (d) cases with drugs sharing the same metabolizing enzymes (DMIs).
- **Symptoms and outcomes**: VAE reports contain also information about the reactions observed in each case, as well as onset dates and intervals (i.e., calculated days from vaccination to onset). We used the MedDRA terms associated with each VAE, as derived by VAERS from the recorded texts describing the symptoms. Symptoms were not processed further, than just removing duplicates. Patient outcomes assessed to be relevant to VAEs were also recorded: death occurrence and date, whether an event was life-threatening, whether a patient required emergency-room or doctor visit, if the patient required hospitalization (its duration and if the stay was prolonged), if the vaccine recipient was disabled as result of the VAE, as well whether the patient recovered. We defined as serious, VAEs for which death, hospitalization, life threatening, or disability events were reported.

Datasets: We extracted VAE data from VAERS, and drug and molecular information from DrugBank.

- **VAERS**: Public data were downloaded on September 6th, 2017. The dataset was last updated on July 17th, 2017 and contained reports processed as of 14th June 2017. The data came in the form of a series of csv files provided by calendar year. In our analysis we considered the full dataset, including also non-domestic (i.e., non US) data.

- **DrugBank**: Public data were downloaded on December 10th, 2017 and came in the form of a single xml file. For analysis we considered only drugs that were annotated as 'approved'. For these drugs we extracted also synonyms, product and international brand names, information about known drug-drug interactions (DDIs), as well as their targets and metabolizing enzymes. We processed further drug target and metabolizing enzyme information by filtering out non-human records. The cleaned dataset also contained no target entries that referred to nucleotides (DNA or RNA) or that were not tagged with a UniProt Id. Last, only drug-target relationships were considered that had pharmacological action explicitly determined to be 'yes'.

Figures:

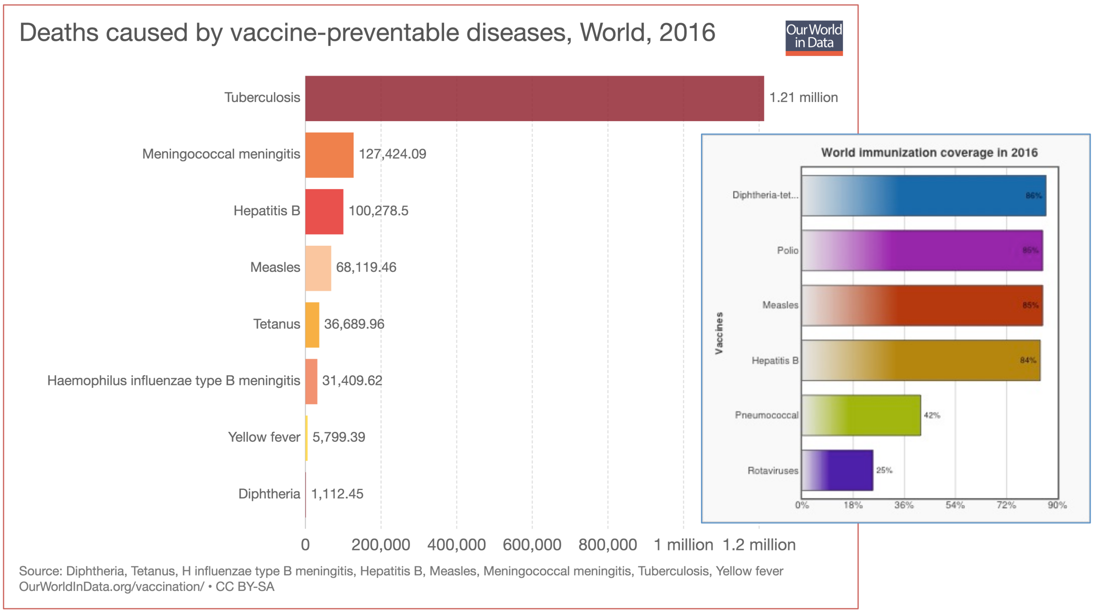

Supplementary Figure 1. ***Vaccine coverage statistics***: right, blue frame: world immunization coverage statistics by the WHO [4]**.** Left, red frame: visualization from Our World in Data [5] regarding the most common and serious vaccine-preventable diseases tracked by the WHO. The WHO has a larger list of 26 diseases for which vaccines are available (including pneumococcal disease, varicella/chicken pox, HPV, Hepatitis A and rotavirus). Also many vaccines are currently in the pipeline of development.

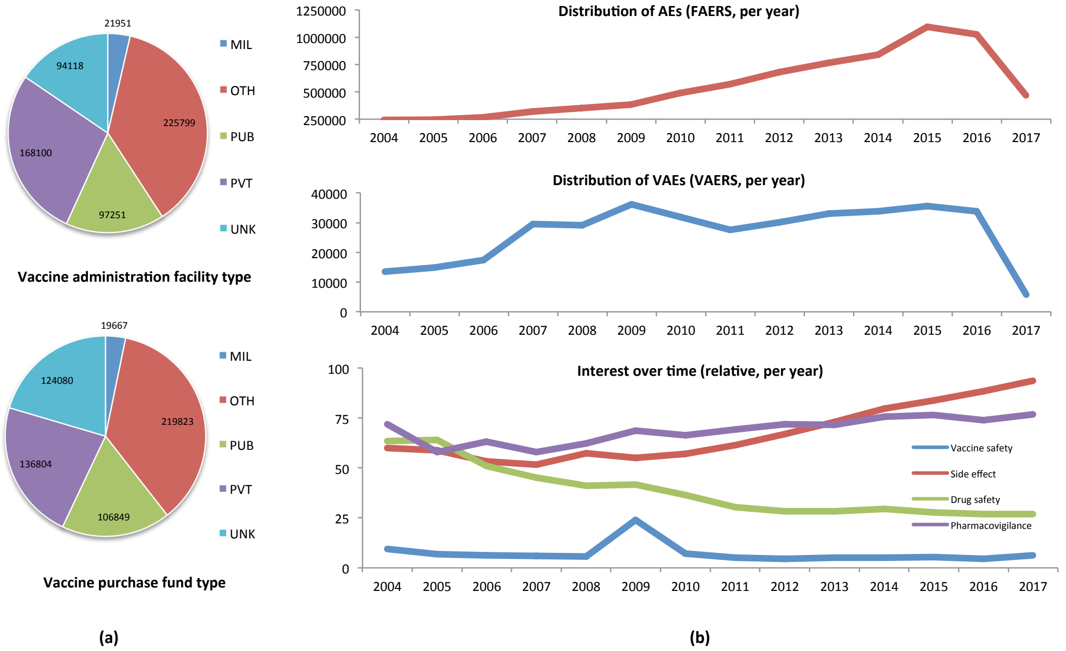

Supplementary Figure 2. ***VAEs - cost management and trends comparison***: a) public spending in VAEs as captured by the types of vaccine administration facility and fund source in VAERS; b) number of cases in FAERS (upper) and VAERS (middle) that happened per year; the timelines are based only on events with available vaccination or onset dates; overall, FAERS contains many more AE cases than VAERS; FAERS contains also few VAEs, probably largely overlapping with VAERS; the decrease in 2016 and 2017 numbers indicates that not all reports had been included yet for those years at the time this analysis took place. AE reporting may be affected by public events as reflected by the similar growth of Google search interests over time for selected terms (lower); the values for every term represent its annual worldwide average search interest relative to its own highest mentioning for the given time frame; a value of 100 represents the peak

popularity for the term, whereas 50 means that the term was half as popular; individual search interests have not been normalized with respect to each others'; characteristically, the term 'Side effect' appears on average at least sixty times more frequently in searches than 'Vaccine safety'; terms 'Adverse effect' or 'Adverse event' were found to be often used in financial contexts and therefore excluded from analysis; data extracted from Google Trends [6].

References

1. Wishart, D. S. *et al.* DrugBank 5.0: a major update to the DrugBank database for 2018. *Nucleic Acids Res.* **46**, D1074–D1082 (2018).
2. Law, V. *et al.* DrugBank 4.0: shedding new light on drug metabolism. *Nucleic Acids Res.* **42**, D1091-1097 (2014).
3. Wishart, D. S. *et al.* DrugBank: a comprehensive resource for in silico drug discovery and exploration. *Nucleic Acids Res.* **34**, D668-672 (2006).
4. WHO | Immunization coverage. *WHO* Available at: http://www.who.int/mediacentre/factsheets/fs378/en/. (Accessed: 2nd April 2018)
5. Vaccination. *Our World in Data* Available at: https://ourworldindata.org/vaccination. (Accessed: 2nd April 2018)
6. Google Trends. *Google Trends* Available at: https://g.co/trends/FDVVW. (Accessed: 2nd April 2018)
